# Production of immunodeficient rabbits by multiplex embryo transfer and multiplex gene targeting

**DOI:** 10.1038/s41598-017-12201-0

**Published:** 2017-09-22

**Authors:** Jun Song, Dongshan Yang, Jinxue Ruan, Jifeng Zhang, Yuqing Eugene Chen, Jie Xu

**Affiliations:** Center for Advanced Models for Translational Sciences and Therapeutics, University of Michigan Medical Center, Ann Arbor, Michigan 48109 USA

## Abstract

Immunodeficient mice have been used predominantly in biomedical research. Realizing that large animal species may have an enhanced ability to predict clinical outcome relative to mice, we worked to develop immunodeficient rabbits by CRISPR/Cas9. We first demonstrated that multiplex embryo transfer efficiently produced multiple lines of single-gene mutant (SGM) founders. Embryos microinjected with single sgRNA targeting *FOXN1*, *RAG2*, *IL2RG* or *PRKDC* were pooled for embryo transfer. As few as three recipients were used to produce twenty SGM founders for four genes. We then demonstrated the powerful multiplex targeting capacity of CRISPR/Cas9. First, two genes on the same chromosome were targeted simultaneously, resulting in three *RAG1/RAG2* double-gene mutant (DGM) founders. Next we microinjected forty-five embryos each with five sgRNAs targeting *FOXN1*, *RAG1*, *RAG2*, *IL2RG and PRKDC*, and transferred them to two recipients. Five founders were produced: one SGM, two DGM, one triple-gene mutant and one quadruple-gene mutant. The present work demonstrates that multiplex embryo transfer and multiplex gene targeting can be used to quickly and efficiently generate mutant rabbit founders. Four lines of SGM (e.g. *FOXN1*, *RAG2*, *IL2RG*, *and PRKDC*) immunodeficient rabbits, as well as multigenic mutant immunodeficient rabbits have been produced. These animals may prove useful for biomedical research.

## Introduction

Immunodeficient mice carrying mutations in genes that are involved in lymphocyte development and/or functions, such as *FOXN1*, *IL2RG*, *RAG1* or *RAG2*, and *PRKDC*, are widely used in biomedical research^1^. Multigenic immunodeficient mice, for example NSG (NOD.Cg-*PRKDC* ^*scid*^ *IL2RG* ^*tm1Wjl*^), NOG (NODShi.Cg-*PRKDC* ^*scid*^ *IL2RG* ^*tm1Sug*^), or NRG (NOD.Cg-*RAG1* ^*tm1Mom*^ *IL2RG* ^*tm1Wjl*^), are particularly useful for allo- and xeno- studies due to their severely compromised immune responses to transplantations^2^. It is realized that large animal species often have an enhanced ability to predict clinical outcome relative to mice^3,4^. Therefore, it is beneficial to develop parallel immunodeficient non-rodent animal models, such as rabbits.

Production of gene targeted transgenic (GTT) rabbits has been a challenge, until recent advent of gene editing nucleases, including ZFN, TALEN, and CRISPR/Cas9 (interchangeably referred to as “Cas9”). These customizable nucleases are efficient in generating double strand breaks (DSBs), which consequently may lead to a functional knockout (KO) of the targeted gene when the DSBs are repaired by non-homologous end joining (NHEJ), or be used to integrate a DNA sequence at a specific locus (e.g. knock-in) through homology directly repair (HDR)^5^. By adapting these nuclease tools, we and others have established efficient platforms for production of KO and knock-in rabbits^6–11^. In 2012, we produced *ApoCIII* KO rabbits using the ZFN approach^11^. In 2013, we generated a number of KO rabbit lines using the Cas9 approach with success rates ranging from 32 to 83%^6^. On average, we used five rabbits (donors and recipients) to produce nine founder KO kits for one target gene^6^.

Given such high efficiency, we reasoned that it is possible to use fewer animals and embryos for production of KO founders. One feasible approach is to pool embryos targeted for different genes for embryo transfer, referred to as multiplex embryo transfer, to reduce the number of recipient animals. We also note that a major advantage of Cas9 mediated gene targeting, the multiplex targeting capacity, has not been fully exploited in GTT rabbit production. Unlike ZFN or TALEN which uses protein motifs for targeting DNA sequences, the Cas9 system finds target sequence by complementation, where a single guide RNA (sgRNA) consisting of complementary sequence to the DNA target is used^12^. The fact that sgRNAs can be easily designed and synthesized makes it possible to use multiple sgRNAs at the same time to achieve multigenic targeting, which has been demonstrated in cells and in transgenic animal production^8,13–15^. This multiplex targeting capacity is particularly useful when the targeted genes are closely positioned on the same chromosome (e.g. *RAG1 and RAG2*).

In the present work, we selected several major immunodeficient-causing genes, *FOXN1*, *RAG1*, *RAG2*, *IL2RG*, and *PRKDC*, to demonstrate the applicability of multiplex embryo transfer and multiplex gene targeting for production of GTT rabbits, and to develop rabbit models of immunodeficiency.

## Results

### *In vitro* validation of sgRNAs

In the first step, we conducted *in vitro* validation of sgRNAs targeting rabbit *FOXN1*, *PRKDC*, *IL2RG*, *RAG1* and *RAG2* (Table 1) using preimplantaional embryos. For each gene, RNA mixture of Cas9 constructs (150 ng/μl Cas9 mRNA plus 6 ng/μl sgRNA) was microinjected into cytoplasm of pronuclear stage embryos. Embryos were cultured *in vitro*, collected at blastocyst stage, and subjected to single embryo PCR using primers described in Table S1 followed by sanger sequencing to identify insertion and deletions (indels) in the corresponding target locus. The indel rates are 87.5% (7/8), 40% (2/5), 50% (6/12), 71.4% (5/7), and 100% (7/7) for *FOXN1*, *PRKDC*, *IL2RG*, *RAG1*, *and RAG2*, respectively. The indel rates (40–100%) are similar to what we reported previously^6^, hence considered acceptable for *in vivo* animal production.

**Table 1:** *In vitro* validation of sgRNA targeting efficiencies.

Chr	Target gene	NCBI Gene ID	Target exon	Target site	PAM	#Embryos sequenced	#Embryos with indels (%)
19	*FOXN1*	100346912	3	GCAATGCTGGGGCTGTGCGG	GGG	8	7 (87.5)
3	*PRKDC*	100125348	1	TTGTCGTGCGGGGACCGCTG	CGG	5	2 (40.0)
X	*IL2RG*	103351862	1	GGAGCCACCCGATCCACTGG	GGG	6	3 (50.0)
1	*RAG*1	100328950	1	GCTGGAGATTGCTCAAGGGA	GGG	7	5 (71.4)
1	*RAG2*	100328951	1	GTGGCTGGGTAACGGAGAGG	AGG	7	7 (100)

Chr: chromosome#.

### Production of immunodeficient rabbits by multiplex embryo transfer

**Figure 1:**
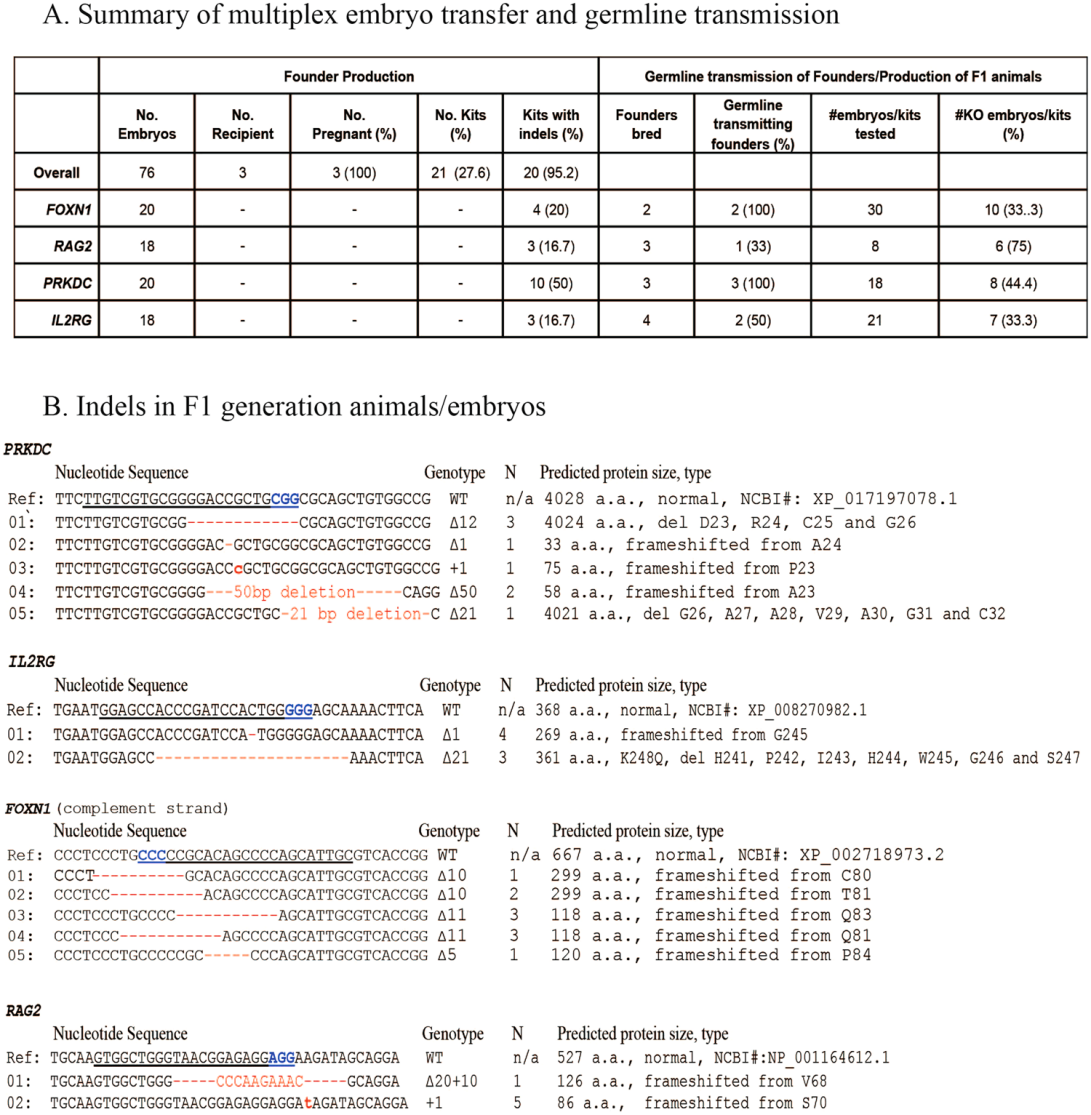
Production of immunodeficient rabbits by multiplex embryo transfer. (**A**) Summary of multiplex embryo transfer results. (**B**) Specific indels identified in F1 generation heterozygous SGM animals. WT: wild type. n: number of animals. n/a: not applicable. a.a.: amino acid. PAM sequences are shown in blue.

Because the indel efficiency for each sgRNA is high, we reasoned that if we pool the embryos targeted for each single gene for embryo transfer, we can use small number of recipients to produce multiple lines of mutant rabbits. To test this, we microinjected embryos with single sgRNA targeting *FOXN1* (n = 20), *RAG2* (n = 18), *PRKDC* (n = 20), or *IL2RG* (n = 18), along with Cas9 mRNA (Fig. 1A). We transferred all 76 embryos to 3 recipients. Twenty-one kits were born, out of which 20 (95%) were confirmed as mutant founders (defined as those carrying indel allele(s)): 4 for *FOXN1* (20%), 3 for *RAG2* (16.7%), 10 for *PRKDC* (50%), and 3 for *IL2RG* (16.7%) (Fig. 1A). Through this work, we showed that as few as three recipients can be used to produce a total of twenty mutant founder rabbits for four different genes.

### Germline transmission of *FOXN1*, *PRKDC*, *IL2RG*, and *RAG2* SGM founders

Upon sexual maturation of single-gene mutant (SGM) founders of *FOXN1*, *PRKDC*, *IL2RG*, and *RAG2*, we bred them with wild-type (WT) counter parts and produced F1 generation kits/embryos. We genotyped the F1 kits/embryos to test if these SGM founders were germline transmitting. Germline transmitting SGM founder(s) were identified for each of the four genes (100%). The percentage of germline transmitting founders ranges from 33% to 100% among different lines. In the F1 generation kits, the percentages of mutant offspring are 33.3% for *FOXN1* (10/30), 44.4% for *PRKDC* (8/18), 33.3% for *IL2RG* (7/21), and 75% for *RAG2* (6/8) (Fig. 1A). These results indicate differential mosaic status of the founder animals.

Unlike F0 founder animals all F1 SGM animals were heterozygous. We performed sequencing analysis to determine the exact mutations in the F1 SGM animals (Fig. 1B). In *FOXN1* F1 mutant animals (n = 10), we identified five types of mutations. In *PRKDC* F1 mutant animals (n = 8), we identified five types of mutations. In *IL2RG* F1 mutant animals (n = 7), we identified two types of mutations. In *RAG2* mutant embryos (n = 6), we identified two types of mutations. These data demonstrate the prevalence of mosaicism in mutant founder animals produced by the CRISPR/Cas9 system. Cautions should be taken for selecting the proper mutations for herd expansion.

### Production of double-gene mutant rabbits of genes on the same chromosome

**Figure 2:**
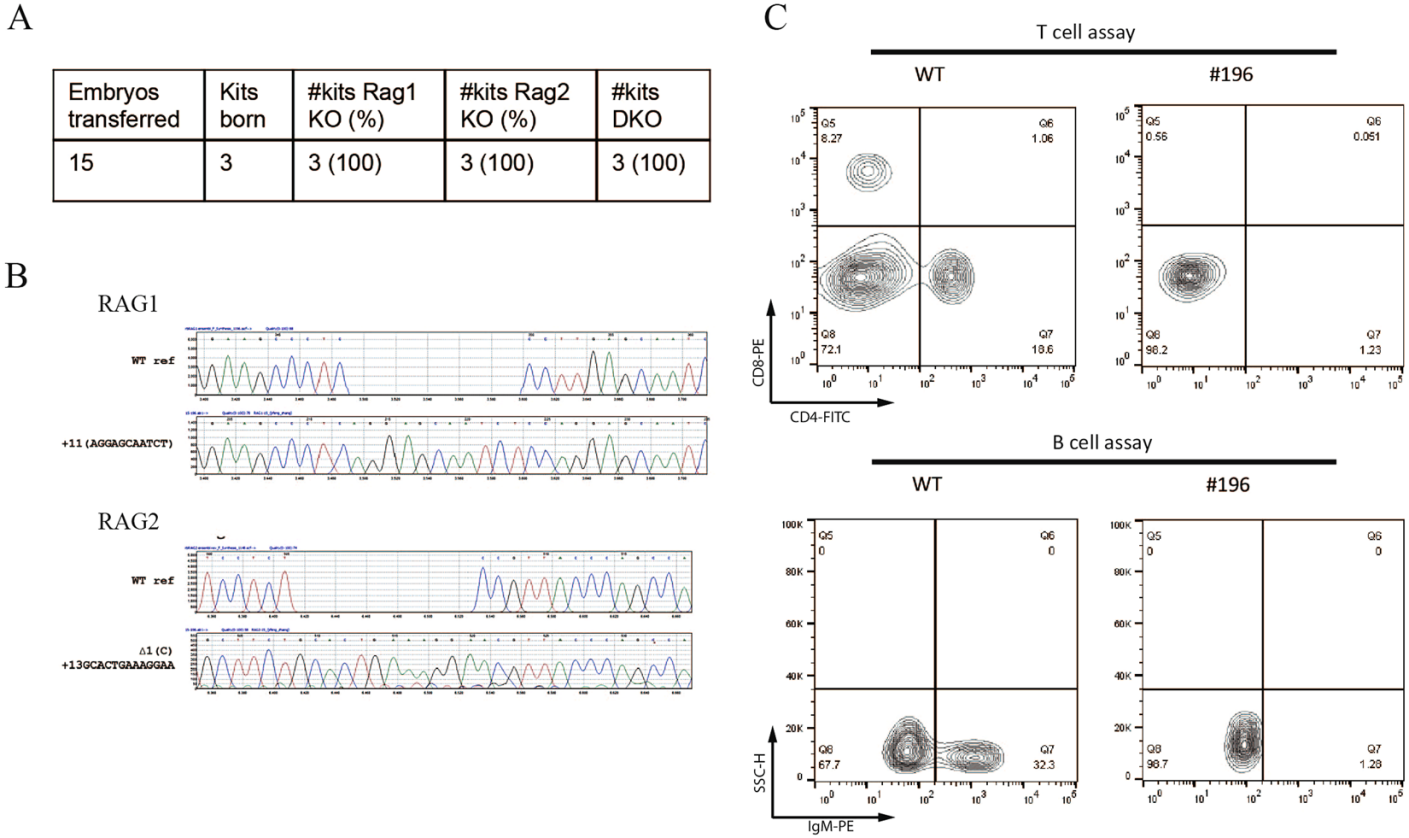
Production of *RAG1*/*RAG2* double knockout rabbits. (**A**) Summary of embryo transfer and genotyping results. (**B**) Homozygous mutations in both *RAG1* and *RAG2* identified in animal #196. (**C**) Flow cytometry analysis revealed defect T- and B-cell populations in *RAG1*/*RAG2* DGM animal #196. WT: wild type.

Next, we worked to exploit the multiplex capacity of Cas9 gene targeting. *RAG1* (location: 175,821,314 to 175,834,519) and *RAG2* (complement, location: 175,840,664 to 175,842,247) genes are adjacent to each other on rabbit Chromosome One, only 6.1 kb apart. We microinjected sgRNAs targeting both *RAG1* and *RAG2* along with Cas9 mRNA to pronuclear stage embryos. Fifteen embryos were transferred to one recipient animal, resulting in 3 kits (Fig. 2A). Genotyping of ear skin biopsies revealed that all 3 kits (100%) carried indels in both *RAG1* and *RAG2* genes (Fig. 2A). In one animal (#196), homozygous bialleic mutations were found for both genes (Fig. 2B); this animal was later confirmed to be deficient of CD4+/CD8+ T and IgM+ B lymphocytes in peripheral blood analyzed by Flow cytometry (Fig. 2C), consistent with reports of *RAG1/2* deficient human patients and *RAG1/2* deficient mice^16^. This work convincingly demonstrates that two genes as close as 6.1 kb apart on the same chromosome can be effectively targeted via CRISPR/Cas9.

### Production of multigenic immunodeficient rabbits

**Figure 3:**
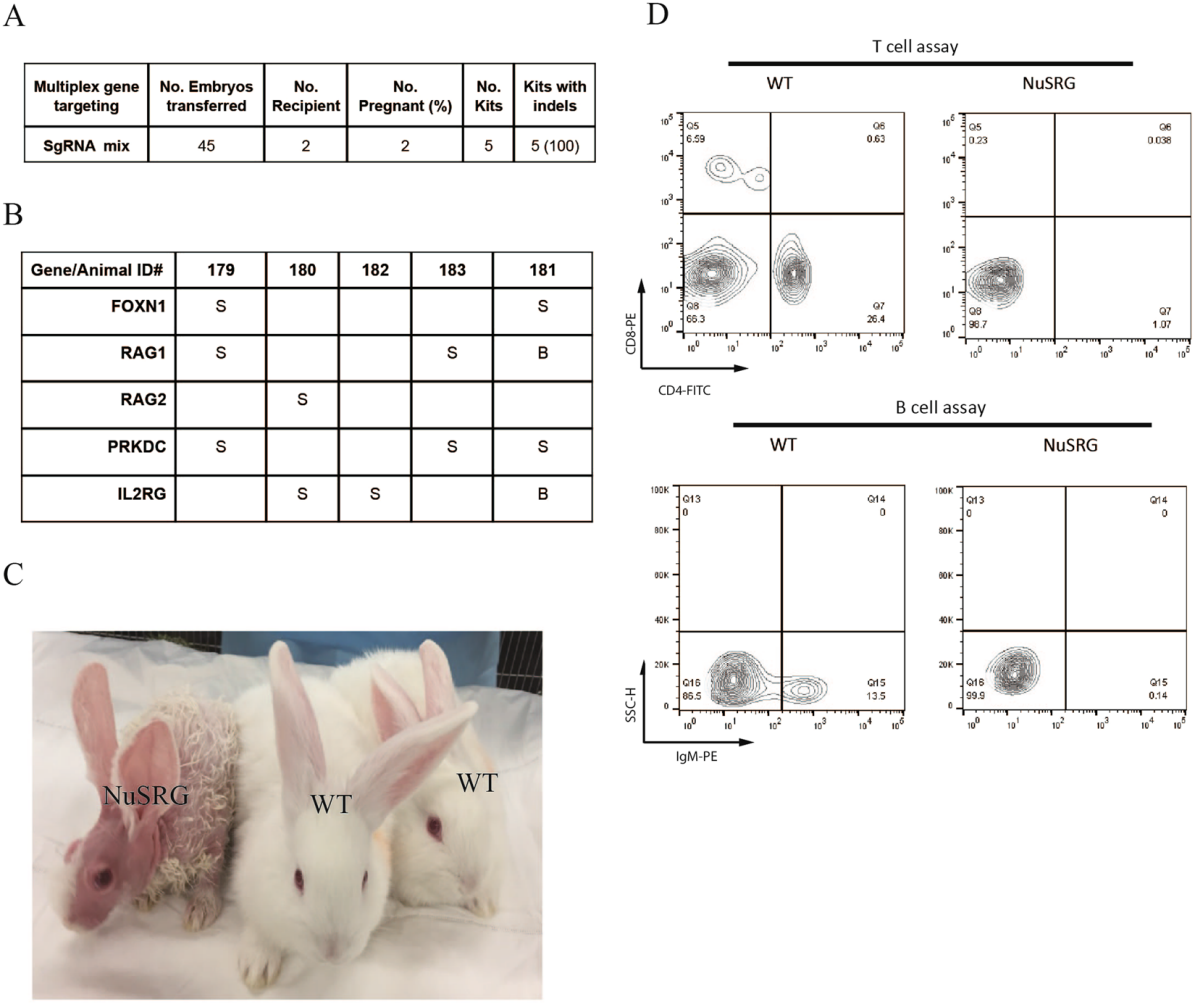
Production of multigenic immunodeficient rabbits by multiplex gene targeting. (**A**) Summary of embryo transfer results. (**B**) Summary of multigenic immunodeficient founder rabbits. S: single allele indel(s). B: bialleic indels. (**C**) NuSRG rabbit (left) and its age-matched WT peers. WT: wild type. (**D**) Flow cytometry analysis revealed defective T- and B-cell populations in the NuSRG rabbit.

We next sought to test if more than two genes can be targeted simultaneously. We pooled sgRNAs targeting all five genes (i.e. *FOXN1*, *PRKDC*, *IL2RG*, *RAG1* and *RAG2*) for microinjection. We transferred 45 such embryos to 2 recipients. Five kits (#179, 180, 181, 182, and 183) were born (Fig. 3A). Genotyping using ear skin biopsies revealed that one animal Kit #182 is a single-gene mutant with indels in *IL2RG*, whereas the remaining 4 kits all carried indels in two or more genes (Fig. 3B). Kit #180 was DGM for *RAG2* and *IL2RG*, whereas Kit#183 was DGM for *RAG1* and *PRKDC*. Kit#179 was triple-gene mutant (TGM) for *FOXN1*, *PRKDC*, and *IL2RG*. Lastly, kit#181 was quadruple-gene mutant (QGM) with indels in *FOXN1* (Nude), *PRKDC* (SCID), *RAG1* (R), and *IL2RG* (G), thus named NuSRG (Fig. 3C). NuSRG rabbit was partially hairless reflecting *FOXN1* KO. Furthermore, it was deficient of T cells and B cells as measured using flow cytometry (Fig. 3D). Similarly, all other 3 multigenic immunodeficient founders (#179, 180, and 183) were also T and B cell deficient as determined by flow cytometry (data not shown). These data show that multigenic immunodeficient rabbits can be efficiently produced via multiplex targeting. We want to point out that due to the lack of robust antibodies for rabbit NK cell markers (e.g. CD16), we were not able to evaluate if NK cells are defective in the NuSRG rabbit as well as other immunodeficient rabbits.

### Off-target events in immunodeficient founder rabbits

One concern with the Cas9 system for gene targeting is the off-target effects. Here we performed off-target analysis for SGM animals. One SGM founder animal was selected for each line (i.e. *FOXN1*, *IL2RG*, *PRKDC*, and *RAG2*). The number of potential off target sites for the sgRNA for each gene is: 57 for *FOXN1*, 6 for *IL2RG*, 15 for *PRKDC*, and 7 for *RAG2*. We selected 18 of the ones that fall within an exon or an intron region of a given gene for analysis: 10 for *FOXN1*, 2 for *IL2RG*, 4 for *PRKDC*, and 2 for *RAG2*. We did not pick any from the ones fall within the intergenic regions as the chances of any such off-target mutations affecting phenotypes are low. Of note, these selected ones included all the ones that fall within an exon or an intron region for *IL2RG*, *PRKDC*, and *RAG2*. For *FOXN1*, there are total 22 potential off-target sites that fall within an exon or an intron region, and we randomly selected 10 for analysis. All 18 selected potential off-target sites were analyzed by PCR followed by sequencing using primers described in Table S2 and BLAST analysis. No off-target mutations were detected (Table 2), indicating that off target mutations are minimal in these founder animals. These animals were then used for herd expansion.

**Table 2:** Off target analysis of founder SGM animals. *ITALIC UPPER CASE LETTERS* are the PAM (NGG) sequences. Lower case letters are the 12 nucleotides adjacent to the PAM sequences. Underlined nucleotides indicate mismatches with the corresponding sgRNA sequence. #mm: number of mismatches. Ins del: insertion or deletion. “no” indicates no off target mutation identified.

Gene/off-target #	Potential off target gene	SgRNA/off-target Sequence	#mm	Ins del
***FOXN1***		GCAATGCTggggctgtgcgg*GGG*	—	—
OT-N-1	NMNAT2	GGCGGCTCggggctgtgcgg*AGG*	7	no
OT-N-2	ZNF831	GGACGTGAggggctgtgcgg*AGG*	6	no
OT-N-3	WDR61	TGAGCGTGggggctgtgcgg*AGG*	6	no
OT-N-4	ZMAT5	TACCGCTCggggctgtgcgg*AGG*	8	no
OT-N-5	RAB11FIP4	AGTGGGGAggggctgtgcgg*AGG*	7	no
OT-N-6	ENSOCUG00000014472	GAGACGCGggggctgtgcgg*TGG*	4	no
OT-N-7	ACO2	GAGTGCCTggggctgtgcgg*TGG*	4	no
OT-N-8	TMEM144	ACAGAAGAggggctgtgcgg*TGG*	6	no
OT-N-9	PLXND1	AGCTTCCCggggctgtgcgg*TGG*	6	no
OT-N-10	ARHGAP25	GGAGGGCAggggctgtgcgg*TGG*	4	no
***IL2RG***		GGAGCCACccgatccactgg*GGG*	—	—
OT-G-1	DVL2	GGCACAATccgatccactgg*AGG*	4	no
OT-G-2	RASGRF1	GCCATCCAccgatccactgg*TGG*	6	no
***PRKDC***		TTGTCGTGcggggaccgctg*CGG*		—
OT-S-1	QARS	GAGACTGAcggggaccgctg*GGG*	6	no
OT-S-2	DCAF1	CCCAGAGAcggggaccgctg*GGG*	8	no
OT-S-3	NAALAD2	AGGGCCCCcggggaccgctg*GGG*	6	no
OT-S-4	ENSOCUG00000010835	TTCTGCCCcggggaccgctg*TGG*	5	no
***RAG2***		GTGGCTGGgtaacggagagg*AGG*	—	—
OT-R2-1	ARHGEF2	TGTTCTGAgtaacggagagg*TGG*	5	no
OT-R2-2	APOBEC1	AAGGCAGAgtaacggagagg*GGG*	4	no

## Discussions

We selected immunodeficient-causing genes *FOXN1*, *PRKDC*, *IL2RG*, *RAG1* and *RAG2* to target in the present study. *FOXN1* encodes a transcription factor required for both hair follicle and thymic development^17^. Deficiency of *FOXN1* leads to hairless and failure of thymus development^18,19^. *PRKDC* encodes a polypeptide that is essential for repair of double strand breaks (DSB) that occurs during somatic recombination of T cell receptor (TCR) and immunoglobulin (Ig) genes^20,21^. Loss of *PRKDC* leads to T and B cell deficiency. *RAG1* and *RAG2* cluster closely on the same chromosome and are key elements for somatic recombination of TCR and Ig genes. Absence of *RAG1* or *RAG2* also results in both T and B cell deficiency^22^. *IL2RG* encodes the common gamma chain protein that is an important signaling component of many interleukin receptors, including those of interleukin −2, −4, −7 and −21^23^. Mutations in this gene cause X-linked severe combined immunodeficiency (X-SCID), characterized by B, T and NK cell deficiency^24^.

The broad applications of immunodeficient mouse models including multigenic ones make the aforementioned genes reasonable targets for us to establish single-gene and multigenic KO immunodeficient rabbits. Prior to the era of gene editing nucleases, production of GTT animals, including knockout and knock-in, are primarily achieved by two methods. In mice and rats where germline transmitting embryonic stem cells (ESCs) are available, genetic manipulations are performed in the ESCs, and the ESCs are used for blastocyst injection to produce founder chimeric animals^25,26^. In non-rodent species where germline transmitting ESCs are not available, genetic manipulations can be performed in somatic cells, followed by using these cells for somatic cell nuclear transfer (SCNT) to produce cloned GTT animals, which is successful in a number of species including cows, pigs, and goats^27^, but not in some other species such as rabbits largely due to the extremely low SCNT efficiencies^28^.

Gene editing nucleases, especially CRISPR/Cas9, have revolutionized the transgenic animal research^12^. Today, as demonstrated in this report, we produced four single-gene mutant founders using only two embryo donor and three embryo recipient rabbits. The number of donors (n = 2) or recipients (n = 3) is actually smaller than the number of mutant genes (n = 4). On average, we used 1.25 animals to produce five mutant founders for a single gene under the multiplex embryo transfer method, a 75% reduction of the number of animals used in our previous report where only embryos targeted for the same gene are pooled to transfer to one recipient (e.g. single gene embryo transfer)^6^. Moreover, there is no compromise in targeting efficiency through multiplex embryo transfer because for each gene multiple founders are produced (ranging from 3 to 10) and germline transmission of mutant alleles is proven. Therefore we suggest researchers consider multiplex embryo transfer provided high targeting efficiencies are validated for each single sgRNA.

We demonstrated the powerful multiplex targeting capacity of the Cas9 system for production of multigenic mutant rabbits. In the first effort, we show that even neighboring genes such as *RAG1* and *RAG2* can be simultaneously targeted. DGM animals can never be produced by conventional breeding of *RAG1* mutant and *RAG2* mutant lines. In mice, such DGM can be achieved by manipulating the ESCs whereas in species like rabbits CRISPR/Cas9 would be the only feasible method for now.

In the second effort, we show that as many as four genes can be targeted at the same time. Production of such complex GTT rabbits will not be possible or at least significantly delayed without using the CRISPR/Cas9 system. The conventional method, based on multiple rounds of gene targeting, is not only time and labor consuming but also limited to mice and rats. The other two gene editing nucleases, ZFN and TALEN, although in theory possess the multiplex targeting capacity, are practically formidable to design, validate and synthesize, and are of substantially lower targeting efficiencies than those achieved by Cas9^29^. It should be noted, however, that most if not all founder animals are mosaic. The mosaicism is more profound in multigenic mutant founders. Therefore careful planning for animal breeding, genotyping, and herd expansion should be implemented. For example, mutations of in frame insertion of deletion should be avoided if a loss of function is the goal of KO, unless such mutations have been known to be causal.

As a more ambitious goal, we aimed to develop immunodeficient rabbit models as general tools for biomedical research. Immunodeficient mice carrying loss of functions mutations in these genes (i.e. *FOXN1*, *PRKDC*, *IL2RG*, *RAG1* and *RAG2*) have been used predominantly in biomedical research, as models for immunodeficient diseases, and recipients for allo- or xeno- transplantations taking advantages of their compromised immune responses^1^. It is realized that large animal species often have an enhanced ability to predict clinical outcome relative to mice^3^. Therefore it is beneficial to develop parallel immunodeficient non-rodent animal models. We consider rabbits for the following reasons: (i) they would enable clinically relevant long-term studies; (ii) many devices, equipment and procedures can be developed and/or tested using these large size animals, a mission often impossible when mouse models are used, for example, testing the safety and efficacy of synthetic blood vessels; and (iii) from a feasibility perspective, such non-rodent animal models should be affordable and laboratory friendly. Rabbit has a short gestation period (30–31 days) and large litter size (4–12/litter). Unlike other large animals such as pigs and monkeys, rabbits can be housed conveniently in an indoor facility in most research institutes hence is economically more affordable.

In the context of immunodeficiency, rabbits may also prove advantageous. For example, the rabbit is a classic animal model for the study of infectious diseases and in particular *S*. *aureus* infections in the lungs^30,31^, which is a major pathogen in human patients; therefore an immunodeficient rabbit model may better reflect *S*. *aureus* infection related pathologies in immunodeficient patients than mouse models do^32,33^. The relatively long lifespan of rabbits over rodents may also prove useful when life expectancy of immunodeficient patients continue to increase. Many therapeutic procedures such as IV infusion can be relatively easily established in immunodeficient rabbits than in their mouse counterparts.

The SGM rabbits we produced in this study can be used for modeling corresponding human primary immunodeficiency diseases: *FOXN1* (OMIM# 600838), *IL2RG* (OMIM# 300400), *RAG2* (OMIM# 601457), and *PRKDC* (OMIM# 600899). Multigenic immunodeficient rabbits may potentially serve as good recipients for engraftment of human cells (e.g. hematopoietic stem cells) or tissues (e.g. fetal liver) to develop humanized rabbit models, which may be useful for the study of a wide spectrum of biomedical subjects, for example, a large animal model with humanized livers for pharmacokinetics and hepatotoxicity studies, vaccines (e.g. *Staphylococcus aureus*) development and testing in a rabbit model with repopulated human lymphocytes, examine long term effects of antiviral therapy of HIV/AIDS on the cardiovascular system, etc. Comprehensive characterization of immunodeficient rabbits and development of these models in various biomedical applications are ongoing and will be reported elsewhere.

*RAG1/2* and *IL2RG* mutant rabbits have been reported by Lai’s group previously using TALEN and Cas9^10,29^. They also produced DGM rabbits of *IL2RG* and *RAG1* by Cas9. Multiplex targeting of more than two genes were tested in rabbit embryos *in vitro*, but no live animals were produced in their study. The present work is consistent with their findings that Cas9 is powerful and capable in generating mutant rabbits. Moving beyond their reports, we demonstrated that two genes on the same chromosome (i.e. *RAG1* and *RAG2*) can be targeted simultaneously, and that live animals carrying indels in up to four genes can be produced. Importantly, we produced nude (i.e. *FOXN1* mutant) and SCID (i.e. *PRKDC* mutant) rabbits, for the first time, in this project. All four lines (*FOXN1*, *PRKDC*, *RAG2*, and *IL2RG*) mutant rabbits were proven germline transmitting and viable F1 generation animals have been produced.

In sum, we have produced multiple SGM and multigenic mutant immunodeficient rabbits using the CRISPR/Cas9 system by multiplex embryo transfer and multiplex gene targeting. High indel rates have been achieved in both approaches. These novel immunodeficient rabbit models, upon full characterization, may be powerful tools for translational biomedical research.

## Materials and Methods

### Animals

New Zealand White (NZW) rabbits were purchased from Covance or Charles River. The animal maintenance, care and use procedures were reviewed and approved by the Institutional Animal Care and Use Committee (IACUC) of the University of Michigan. All procedures were carried out in accordance with the approved guidelines.

### Single guide RNA design and CRISPR/Cas9 construction

Plasmids used in this study (Cas9 expression plasmid JDS246 and sgRNA expression plasmid DR274) were purchased from Addgene. sgRNAs were designed using Zifit software (http://zifit.partners.org/ZiFiT/), synthesized and cloned into the plasmid DR274 respectively. Sequences of individual sgRNAs are shown in Table 1.

### RNA synthesis

Cas9 mRNA was *in vitro* transcribed, caped and polyadenylated using the T7 mScript™ Standard mRNA Production System (C-MSC100625, CELLSCRIPT, Madison, WI). sgRNAs were *in vitro* transcribed by using T7-Scribe™ Standard RNA IVT Kit (C-AS3107, CELLSCRIPT). Cas9 mRNA and sgRNAs were diluted in RNase-free TE buffer (1 mM Tris-Cl pH 8.0, 0.1 mM EDTA), stored in −80 °C in 10 μl aliquots, and were thawed and kept on ice before microinjection.

### Embryo microinjection and transfer

Embryos collection, microinjection and transfer were performed as previously described^6^. Briefly, sexually matured NZW female rabbits were superovulated by subcutaneous injection of follicle-stimulating hormone (FSH, Folltropin-V, Bioniche Life Sciences, Canada) twice per day (3 mg for the first two injections, 5 mg for the next two injections and 6 mg for the last two injections). Seventy-two hours after the first FSH injection, a single dose of 200 IU human chorionic gonadotropin (hCG, Chorulon, Intervet, Holland) was intravenously administered to induce ovulation. The donor females were mated with male rabbit immediately after hCG injection. At the same time, sexually matured recipient female rabbits was synchronized by injection of 0.3 mL Gonadotropin-releasing hormone (GnRH) agonist (Receptal®, Merck animal health) intramuscularly. Eighteen hours post insemination (psi), the donor rabbits were euthanized. The oviduct ampullae were recovered, flushed with 10 mL of Hepes buffered manipulation (HM) medium containing 25 mM Hepes buffered TCM 199 (#12350039, Life Technologies, Grand Island, NY) supplemented with 10% fetal bovine serum (FBS, #12003 C, Sigma, St. Louis, MO), and the recovered embryos were observed under a microscope for the occurrence of fertilization, and then kept in the HM medium at 38.5 °C in air.

Microinjection was performed on pronuclear stage embryos 19–21 h psi using a micromanipulator under the inverted microscope equipped with a differential interference contrast (DIC) device. Rabbit embryo was held with a holding glass pipette (120–150 μm diameter) in HM medium. A mixture containing 100 ng/μL Cas9 mRNA and 6 ng/μL sgRNA were used for cytoplasm microinjection. Injected embryos were washed three times in embryo culture medium, which consisted of Earle’s Balanced Salt Solution (E2888, Sigma) supplemented with non-essential amino acids (M7145, Sigma), essential amino acids (B-6766, Sigma), 1 mM L-glutamine (25030–081, Life Technologies), 0.4 mM sodium pyruvate (11360-070, Life Technologies) and 10% FBS. Twenty to thirty injected embryos were surgically transferred into the oviduct of a synchronized recipient doe on the same day. For *in vitro* validation, instead of transferring to recipient doe, the injected embryos were cultured *in vitro* for additional 3–4 days until they reach blastocyst stage.

### Confirmation of gene targeting events

To evaluate the gene targeting efficiency *in vitro*, *in vitro* cultured blastocyst stage embryos were lysed individually and genomic DNA was extracted. To get better PCR reaction, the whole genome was replicated using a REPLI-g® Mini Kit (Qiagen, Germantown, MD) following the manufacturer’s protocol with slight modification. Briefly, for harvesting denatured DNA, 3.5 μL Buffer D2 was added to each embryo, mixed by vortexing and centrifuged briefly. The samples were incubated on ice for 10 min. After that 3.5 μL Stop Solution was added, mixed by vortexing and centrifuged briefly. For replication, 2 μL of the denatured DNAs were added to 8 μL master mix and incubate at 30 °C for 10–16 h. Then REPLI-g Mini DNA Polymerase was inactivated by heating at 65 °C for 3 min. To determine genotypes of founder animals, we extracted genomic DNA from ear skin tissues biopsied. Genomic DNAs were used for PCR amplification with corresponding primer sets (Table S1). PCR products were purified and sequenced for detection of indel mutations proximate to the sgRNA target sequence. On the chromatographic curves, peaks on peaks approximate the target site indicate indel events. Mutation Surveyor (Softgenetics, State College, PA) was used to analyze sequencing results. Animals carrying indel alleles were identified as KO founders. It should be noted however, not all indels will lead to loss of function of the targeted gene; we used KO in this text to refer to all indel events for simplicity purposes.

### Germline transmission

To identify if a founder rabbit is germline transmitting the KO allele, the founders were bred with WT rabbits respectively. We sampled the ear skin tissues from each kit or the derivative embryos for genotyping as described previously.

### Off-target analysis

To analyze the off-target effects we used BLASTn to identify the exact match to a 15 nt sequence: the 12 nt of each sgRNAs adjacent to PAM with all four possible PAM sequences NGG (AGG, TGG, CGG and GGG). Considering the fact that mutations in exon or intron regions are more likely to cause genetic and phenotype changes than those in the intergenic regions, we analyzed randomly selected potential off-target loci that fall within exon or intron regions for each gene. We did not analyze the ones that fall onto intragenic regions. Primers (Table S2) were designed to amplify the potential off-target sites using the genomic DNA isolated from the animals. The PCR products were sequenced (using the sequence primers showed in Table S2) and blasted to detect any off-target events. We tested one animal for each of the single gene KO line.

### Fluorescence-activated cell sorting (FACS) analysis of T and B lymphocytes development

To analyze T and B lymphocytes in whole blood, approximately 1 mL peripheral blood was collected from the rabbit central ear artery to a heparin coated tube. The red blood cells were lysed using ammonium chloride lysis buffer which consisted of 0.16 M ammonium chloride and 0.17 M Tris, adjusted PH to 7.2. FITC-conjugated anti-rabbit CD4 (KEN4, IgG2a), anti-rabbit CD8 (12.C7, IgG1) and anti-rabbit IgM (NRBM, IgG1), were purchased from Bio-Rad. Anti-mouse IgG-phycoerythrin (PE) was obtained from Santa Cruz (CA, USA). About 0.5 × 10^6 to 1 × 10^6 cells per sample were incubated with primary antibody in 100 μL FACS buffer (PBS pH7.4, 0.1% BSA, 0.05% NaN3) for 30 min on ice, washed twice by centrifugation at 300 g for 10 min with ice-cold FACS buffer, and then incubated with a labeled second antibody under the same conditions. After washing, the cells were resuspended with 300 μL ice-cold FACS buffer before FACS analysis. At least 10,000 live cells were used for the Flow cytometry analysis using MoFlo Astrios cell sorter (Beckman). FlowJo software v10 (Tree Star, Ashland, OR, USA) was used to analyze the FACS data.

## Electronic supplementary material

Supplementary information
