# Supplementary material for: Production of immunodeficient rabbits by multiplex embryo transfer and multiplex gene targeting
Source: Sci Rep. 2017 Sep 22;7:12202. doi: 10.1038/s41598-017-12201-0 (PMC5610260; doi:10.1038/s41598-017-12201-0)
Supplement: Supplementary file 1 — Supplementary information [file 41598_2017_12201_MOESM1_ESM.pdf]

Title

Production of immunodeficient rabbits by multiplex embryo transfer and multiplex gene targeting

Authors

Jun Song[1], Dongshan Yang[1], Jinxue Ruan, Jifeng Zhang, Yuqing Eugene Chen*, Jie Xu*

Supplementary information

Table S1. Primers used for genotyping.

Gene/Primer Name	Sequence (5’ to 3’)
Rag1	
rbRag1_F	AATGCCTCTGTTCTCCGTTCTATGAT
rbRag1_R	AAGGCCTTGGGTTTTACTGTCC
Rag2	
rbRag2_F	GCGGGAAGGTTCTGGGTCTCT
rbRag2_R	AACATGGGGCAGGCAATCAGC
Prkdc	
rbPRKDC_F	TCGCGGGAAATCTGGGGAGTA
rbPRKDC_R	GGGGGAGCAGAAGGACAAATCAC
Il2rg	
rbIL2RG_F	CCCCCACCCCCACCTCCTCTG
rbIL2RG_R	CCCCCTTCTCAATCCACTCCCCTACT
Foxn1	
rbFOXN1_F	CCGACCCCAGGCCCCAGAAGTGT
rbFOXN1_R	GCAGGGAAGCCAGGCAGGAAGG

Table S2. Primers used for off-target analysis.

Gene, off-target#/Primer Name	Sequence (5' to 3')
Foxn1, OT-N-1	
OT-N-1-F	TGGCCAGCTCACGCACAGT
OT-N-1-R	TGAAATCTCCGACGGTTCTCTTA
OT-N-1-seq	GCCCACAGGAACAGCGAAGC
Foxn1, OT-N-2	
OT-N-2-F	AGTGGGTGGGTGAGCGTGAGC
OT-N-2-R	GCCAGGGCCGGGTAAGTAAGG
OT-N-2-seq	GCGTCTTCTCCCAGGTCTCAA
Foxn1, OT-N-3	
OT-N-3-F	CGTTGCGCTGCTGTCCACTC

OT-N-3-R	GATCTTGATGTAGCCGTCGTCTGA
OT-N-3-seq	ACAGAGGACTGGGCAGGGAAGG
Foxn1, OT-N-4	
OT-N-4-F	GTCCCTTCCTCGTGGTCCATT
OT-N-4-R	GCACCGACCTCCTGCAAGCT
OT-N-4-seq	CGCCACCTCGACCTGTGACTT
Foxn1, OT-N-5	
OT-N-5-F	CCCTACCTCCGACGCTTGTCT
OT-N-5-R	CGCTGGCTGAAGGAATGGAC
OT-N-5-seq	AAAGGTTCGCGGGCGGACTG
Foxn1, OT-N-6	
OT-N-6-F	GACGCCTCGCTGTCGGTCAT
OT-N-6-R	GGGTTGTGGGTGTTGGGAAGT
OT-N-6-seq	TTGGGAAGTGAACCAACAGATGGAA
Foxn1, OT-N-7	
OT-N-7-F	GAGGAGTAGAGGTCCGAGGTAA
OT-N-7-R	AGGGTAAGAGGCCGGTTCA
OT-N-7-seq	AGGTGGCCTAGTCCCAGACG
Foxn1, OT-N-8	
OT-N-8-F	GCCAACACAGAAACCCCCTCCCTCATT
OT-N-8-R	CACCCAGCCCGCTCCCATCCA
OT-N-8-seq	AAACTTCAGGGAAGGCTGTG
Foxn1, OT-N-9	
OT-N-9-F	CTCCCTGGGCCGTAGTGAGTGT
OT-N-9-R	CCCTGCCGAGATCCTTTGGTC
OT-N-9-seq	TTGGTCGGTCACAAGCAGGAA
Foxn1, OT-N-10	
OT-N-10-F	GGGGGGCCTGGAGAACACAT
OT-N-10-R	CCTTGGCCCAGACATCCTTAGAGA
OT-N-10-seq	ACATGGCCTTCTTGGCTTGA
Il2rg, OT-G-1	
OT-G-1-F	TTCCAACTGCCGCCAACA
OT-G-1-R	CGGGGAGACGAAGGTGATTTAC
OT-G-1-seq	TGGATCAGGATTTCGGGTGA
Il2rg, OT-G-2	
OT-G-2-F	TGGCGGAGCAGCGTGTCAGG
OT-G-2-R	TGGCCAGCTTTTCAGGAGGGAGTA
OT-G-2-seq	TTATGGCCTTCACAGGGTTGC
Prkdc, OT-S-1	
OT-S-1-F	TGTATGGCGTGGCTTCCCGACTCAGG
OT-S-1-R	GCCATGGCCAGGGAAAGACTCACAGC
OT-S-1-seq	ACCACATTCCTGCTCAAAGTCC
Prkdc, OT-S-2	

OT-S-2-F	CAGCCCTTCCCTCCCAGAT
OT-S-2-R	GTGCCTCAAAGCCGATACTT
OT-S-2-seq	GCAGGCTTAGTTCTGGCACAT
Prkdc, OT-S-3	
OT-S-3-F	GCAGAGCCCAGATTCCGCAGTT
OT-S-3-R	GCAGCCAAGCACATCCAAAGGTA
OT-S-3-seq	GTGAGCAGCCTCGGACCTT
Prkdc, OT-S-4	
OT-S-4-F	ACTTCTTAGGCGGAGGAACAGAGC
OT-S-4-R	CAGGGGTCACCGAGGGGTT
OT-S-4-seq	CCCCTGGGTAGGGTCTTCACG
Rag2, OT-R2-1	
OT-R2-1-F	CCGCCTTTGCTTTTCGCTCCAGTT
OT-R2-1-R	CCCCTCCCAAAGACCCGCAGTTC
OT-R2-1- seq	TTTCCTTTCCCAAACCCTAACC
Rag2, OT-R2-2	
OT-R2-2-F	TCAGACACAGAGGAAAACAAAAA
OT-R2-2-R	TGGGAGACAGCAAATGATGG
OT-R2-2-seq	GACCTAAGCCTGGGTGTT
